# Preparation of hollow TiO_2_ nanoparticles through TiO_2_ deposition on polystyrene latex particles and characterizations of their structure and photocatalytic activity

**DOI:** 10.1186/1556-276X-7-646

**Published:** 2012-11-24

**Authors:** Jingang Wang, Jiemei Yu, Xiaoli Zhu, Xiang Zheng Kong

**Affiliations:** 1 College of Chemistry and Chemical Engineering, University of Jinan, Jinan, 250022, China

**Keywords:** Nanostructured inorganic materials, Surfaces, Interfaces, Sol-gel processes, PSt/TiO_2_ core-shell composites, Preheating, Calcination, Photodegradation catalysis

## Abstract

In a mixed solvent of water and ethanol, polystyrene/titanium dioxide (PSt/TiO_2_) composite particles of core-shell structure were prepared by hydrolysis of tetrabutyl titanate in the presence of cationic PSt particles or anionic PSt particles surface-treated using γ-aminopropyl triethoxysilane. Hollow TiO_2_ particles were obtained through calcination of the PSt/TiO_2_ core-shell particles to burn off the PSt core or through dissolution of the core by tetrahydrofuran (THF). An alternative process constituted of preheating the PSt/TiO_2_ particles at 200°C to allow partial crystallization followed by calcination or PSt dissolution by THF. The outcome TiO_2_ particles thus prepared were examined by TEM, and hollow TiO_2_ particles were observed. The crystalline phase structure and phase transformation were characterized, which revealed that preheating before the removal of the PSt core was useful to achieve the desired hollow TiO_2_ particles, and the calcination process was beneficial to the formation of anatase and rutile structures. The tests of TiO_2_ particles as catalyst in the photodegradation of Rhodamine B demonstrated that a much higher catalytic activity was observed with the TiO_2_ hollow particles prepared through calcination combined with preheating.

## Background

With constant increase in the volume and number of organic pollutants, establishment and applications of practical and effective processes for the degradation of these pollutants have been in urgent need. Nanoparticles of titanium dioxide (TiO_2_) have been extensively studied owing to their high catalytic activity, nontoxicity, cost effectiveness, and high stability. In the preparation of TiO_2_ particles, it is particularly crucial to achieve the desired structure and crystallinity because they impose a great impact on the particles' catalytic activity. Hollow TiO_2_ particles, besides their high performance as a catalyst, are also characterized by their high specific surface, high permeability, high strength, and low density [1,2]. The common practice in the preparation of hollow TiO_2_ particles has been to use a template. The process involves, in a first step, deposition of a TiO_2_ coat on the surface of inorganic or organic particles [3,4] as the template, leading to the formation of bi-component core-shell composite particles with the shell consisting of TiO_2_, and followed by removal of the template core material through dissolution by a solvent or through calcination to burn off the core material. Due to the easy hydrolysis of the primary or the precursor materials used to produce TiO_2_, deposition of a smooth and homogeneous coat on a desired surface is often highly challenging. The selection of the template core material is therefore essential. Up to date, colloidal carbon spheres [2,4-6], originated from glucose, have been often used for this purpose. The surface of these carbon spheres is hydrophilic and possesses -OH and -C=O groups, which renders the surface of the template microspheres directly workable. The fabrication of the template microspheres is quite easy. The size of the microspheres is, however, hard to adjust. It is well known that the size of polymeric particles, particularly those prepared through emulsion polymerization, is easily adjustable. Polystyrene (PSt) latex particles have been used for this purpose [7-10]. Nevertheless, PSt is highly hydrophobic and its compatibility with TiO_2_ is quite poor. A special treatment for PSt particle surface is often needed in order to achieve the desired TiO_2_ deposition. Surface sulfonation of PSt particles is one common practice, which has been effected either through a chemical treatment for the PSt particles [8] by concentrated sulfuric acid or through copolymerization of styrene (St) with a functional comonomer, sodium *p*-vinylbenzene sulfonate [9-11] for instance. The surface of PSt particles thus prepared are negatively charged, TiO_2_-coated PSt composite particles and hollow TiO_2_ particles were effectively prepared this way. It is well accepted that TiO_2_, particularly its precursor, when prepared from the hydrolysis of tetrabutyl titanate (TBT or titanium tetrabutoxide), is negatively charged, and it is often believed that positively charged PSt particles would facilitate the deposition of TiO_2_ on their surface, thanks to an enhanced electrostatic attraction between the negatively charged TiO_2_ precursor and the positively charged particle surfaces [12-15]. Preparation of cationic polymer particles through emulsion copolymerization of vinyl monomer with a functional cationic monomer has been reported to be an effective pathway to achieve polymer/TiO_2_ core-shell particles [7], though the availability of cationic monomers for this purpose is very limited in comparison with anionic functional monomers. In addition, the stability of the cationic latexes thus prepared is often poor.

γ-Aminopropyl triethoxysilane (KH550) is a known alkoxy silane coupling agent. Its terminal amine group can form strong association with specific groups, its ethoxy groups are easy to hydrolyze in water to deliver hydroxyl silane, and a condensation reaction will occur between the hydroxyl silane molecules themselves or between these hydroxyl groups and those from different molecules [16]. These reactions also allow bridging-up between inorganic particles such as SiO_2_, TiO_2_, and organic compounds [17]. KH550 was thus used in the preparation of porous SiO_2_ particles [18-20] as a coupling agent between PSt particles and silica. Peng et al. [21] reported a process for the fabrication of superhydrophobic conductive coatings through modification of carbon nanotubes by an organic-inorganic hybrid material, obtained also with KH550 as the coupling agent between silica and an amphiphilic copolymer.

In this paper, both cationic and anionic PSt latex particles with uniformed size were prepared through emulsion polymerization, and the anionic ones were further subjected to surface modification using KH550 with the purpose to convert the anionic surface charge to cationic. Using both types of PSt latex particles as the templates, PSt/TiO_2_ core-shell composite particles were achieved through hydrolysis of TBT in the presence of PSt particles. Hollow TiO_2_ particles were then obtained by removal of PSt core from the composite core-shell particles by dissolution or calcinations. Different protocols were used for the process, and the outcome TiO_2_ particles were characterized, followed by their tests of catalytic activity in the photodegradation of Rhodamine B. A higher catalytic activity was observed when the TiO_2_ hollow particles prepared through calcination combined with preheating were used as the catalyst.

## Methods

### Materials

All materials are domestic products of China. Styrene and methacrylic acid (MAA), both purchased from Guangcheng Chemical Reagents Co. Ltd., Tianjin, were distilled under reduced pressure before use to remove the inhibitors. Potassium persulfate (KPS; Fuchen Chemicals Reagent Factory, Tianjin), TBT (Kermel Chemical Reagent Co. Ltd., Tianjin), ethanol (EtOH; Guangcheng Chemicals Reagents Co. Ltd.), tetrahydrofuran (THF), and 2,2-azobis(2-amidinopropane) dihydrochloride (AIBA; Beijing Institute of Chemical Engineering) were all analytical grade and used as received. Alpha olefin sulfonate (AOS; Jilida Chemical Co. Ltd., Hangzhou), polyvinylpyrrolidone (PVP; K-30, Sinopharm Chemical Reagent Co. Ltd., Shanghai), and γ-aminopropyl triethoxysilane (KH550; Yijia Chemicals of Jinan), all industrial grade, were used as received. Methacryloxyethyl hexadecyl dimethyl ammonium bromide (DMHB) was prepared in the laboratory as previously described [22]. Rhodamine B (RB), analytical grade, was purchased from Sinopharm Chemical Reagent Co. Ltd.

### Preparation of PSt particles

Both cationic and anionic latex particles were prepared. For the cationic latex preparation, 1.5 g of cationic monomer DMHB was added to a 500-mL four-necked round-bottom flask equipped with an overhead mechanical stirrer, a N_2_ inlet, and a coiled Graham condenser. Under constant N_2_ flow in and out, double-distilled water (240.0 g) was added into the flask followed by 28.5 g of St under stirring. After the content in the flask was stirred for 20 min, the flask was placed into a water bath with temperature set at 70°C and stirred at 250 rpm for 15 min, followed by addition of 30 mL of AIBA aqueous solution of 0.25 wt.% concentration to initiate the polymerization. A cationic PSt latex with 9.60 wt.% solid was thus obtained at the end of the 8-h polymerization. For the preparation of anionic latex particles, the whole process was kept the same as that for cationic latex preparation except for the following: 0.1 g of anionic AOS was used instead of cationic DMHB, monomer St was replaced by a mixture of St and MAA, and anionic initiator KPS was used instead of cationic AIBA. The polymerization was allowed to proceed for 12 h to have anionic latex with solids of about 9.40 wt.%.

### Preparation of PSt/TiO_2_ core-shell particles

To prepare PSt/TiO_2_ core-shell particles, experiments were carried out using two types of cationic PSt particles from different processes. One was prepared using DMHB, the cationic monomer; the other was prepared using the anionic particles after surface modification by KH550. When using cationic PSt particles, 5 mL of the cationic as-prepared PSt latex was dispersed into 50 mL of EtOH. A solution of 1 mL of TBT in 10 mL of EtOH was added into the mixture of PSt latex and EtOH within 10 min at constant rate under vigorous agitation; the reaction was continued for another 2 h under stirring at 150 rpm. The whole process was carried out at 30°C.

Concerning the process using anionic PSt particles after surface modification by KH550, 5 mL of anionic PSt latex (of 9.40 wt.% solids) and 0.10 g of KH550 were first added into 50 mL of EtOH; the mixture was stirred for a few minutes and followed immediately by addition of a TBT (1 mL) solution in EtOH (10 mL) within 10 min at a constant rate under vigorous agitation. Stirring at 150 rpm was continued for another 2 h at 30°C upon the completion of the TBT solution addition. To get a better dispersion, a solution of 0.5 g of PVP K-30 in 5 mL of EtOH was also added about 10 min before the end of the process in order to provide the particles with a better stability.

### Preparation of hollow TiO_2_ particles

To prepare the hollow TiO_2_ particles, the dispersion sample of PSt/TiO_2_ core-shell particles in EtOH was subjected to centrifugation at 6,000 rpm for 10 min. The centrifugate was redispersed into THF, and the mixture was kept stirred at 30°C for 4 h to dissolve the PSt core out. The sample was centrifuged again. The resultant sample was denoted as sample D, referring to the dissolution process. Instead of the THF dissolution in sample D, for sample C (referring to the calcination process) preparation, the centrifuged PSt/TiO_2_ sample was subjected to EtOH washing and drying up at 50°C, followed by calcination at 600°C for 4 h to burn off the PSt core. Alternatively, the original dispersion of PSt/TiO_2_ core-shell particles was put through preheating at 200°C for 6 h with the objective to achieve a partial crystallization in TiO_2_, the shell materials. The same processes of THF dissolution or calcination were carried out following the preheating. The corresponding samples thus prepared were denoted as sample HD (for preheating plus dissolution by THF) and sample HC (for preheating plus calcination).

### Instruments and characterization

Zeta potentials of different PSt particles were determined using Malvern Nano-ZS (Malvern Instruments, Worcestershire, UK). The size and morphology of all particles at different steps were examined using transmission electron microscopy (TEM; JEM-2010, Tokyo, Japan). Crystal structure of the final hollow TiO_2_ particles was characterized by X-ray powder diffraction (XRD; D/Max-RA, Rigaku, Tokyo, Japan). Part of the samples was also put through differential thermal analysis (DTA) and thermal gravimetry analysis (TGA) using Diamond TG/DTA (PerkinElmer, Waltham, MA, USA).

### Tests on catalytic activity of TiO_2_ particles in RB photodegradation

Catalytic activity of different as-prepared TiO_2_ hollow particles was tested in RB photodegradation and compared with each other. In 200 mL of RB solution (pH 4.0) with a concentration of 5 mg/L, 0.02 g of TiO_2_ powder was added; the solution was ultrasonicated for 5 min, and the degradation of RB was effected under radiation of a high-pressure mercury lamp (250 W; Tianjin Yingze Technology Co. Ltd., Tianjin, China) under constant stirring. A sample was taken out every 10 min, the supernatant of each sample was collected after high-speed centrifugation, and its absorbance was determined using ultraviolet-visible spectrophotometer (Lambda 35, PerkinElmer) at 554 nm in order to estimate the residual RB concentration.

## Results and discussions

### Zeta potential of PSt particles

Zeta potentials of all the three types of PSt particles, i.e., cationic, anionic, and those with surface modified by KH550, were determined. The results showed that the cationic PSt particles prepared with cationic DMHB were characterized with a positive zeta potential of +19.9 mV, and the anionic PSt particles prepared with anionic monomers AOS and MAA showed a negative potential of −48.3 mV. Also, this negative zeta potential turned positive (+10.8 mV) once the dispersion of these anionic PSt particles was treated with KH550. KH550 (molecular formula H_2_NCH_2_CH_2_CH_2_Si(OC_2_H_5_)_3_) is a coupling agent with a terminal group of amine. When modifying the surface of negatively charged PSt particles by adding KH550 in the dispersion of PSt particles in the mixed solvent of EtOH and water, it is believed that an electrostatic attraction between the positively charged KH550 and the anionic groups of COO^−^ and -SO_3_^−^ on the surface of PSt particles promotes the adsorption of KH550 on the particles. The abrupt switch of zeta potential from −48.3 mV (anionic PSt particles) to +10.8 mV (the same PSt particles treated with KH550) was seen as a solid support that anionic PSt particles were successfully converted to cationic ones through electrostatic adsorption of KH550 onto the surface of the anionic PSt particles.

### Function of KH550 in the formation of PSt/TiO_2_ core-shell structure

**Figure 1:**
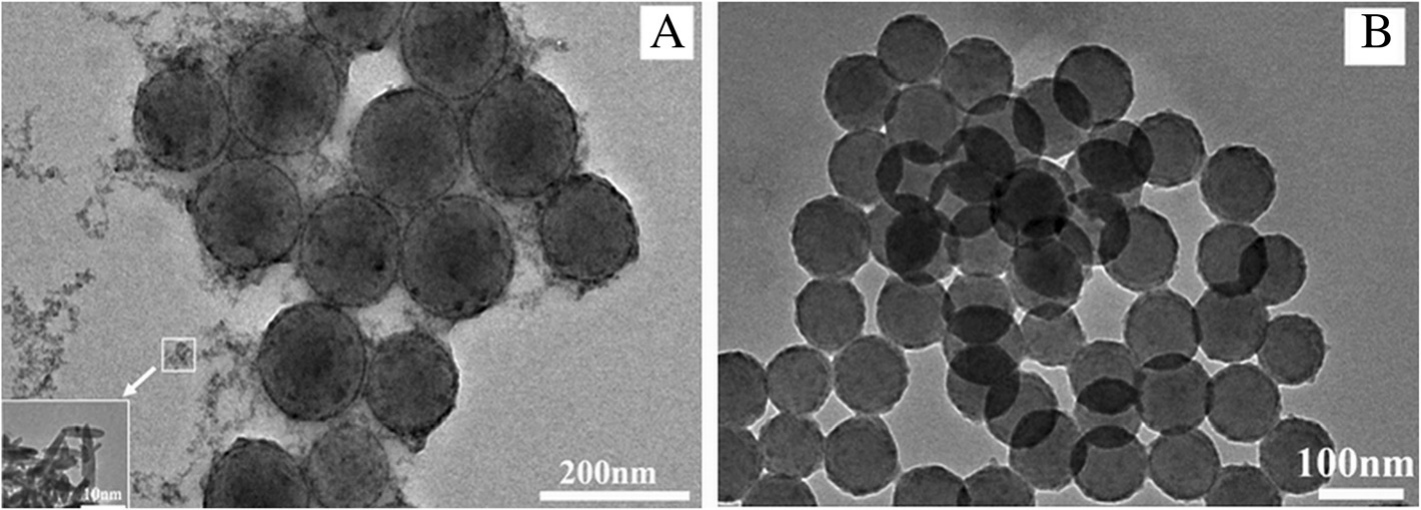
**TEM images of PSt/TiO_2_ core-shell particles.** Particles obtained through TiO_2_ deposition onto cationic PSt particles prepared with cationic DMHB monomer (**A**) and TiO_2_ deposition onto anionic PSt particles with surface charge converted by KH550 treatment (**B**).

It is commonly known that TBT is prone to quick hydrolysis. In the presence of PSt particles, the *in situ* produced TiO_2_ tends to nucleate to form new crystals, and its deposition on PSt particles is limited. Knowing that the precursor molecules in TiO_2_ formation are rich with hydroxyl groups and are negatively charged, it is believed that TiO_2_ deposition on PSt particles is enhanced when PSt particles are surface-modified to become more hydrophilic, or positively charged, which has been used for the preparation of PSt/SiO_2_ core-shell particles [19]. In this work, cationic PSt particles obtained using DMHB and those after surface modification by KH550 were both used to prepare PSt/TiO_2_ core-shell particles. Their TEM micrographs are given in Figure 1.

In Figure 1A are shown the PSt/TiO_2_ particles prepared with cationic PSt particles using DMHB, from which one can see that most of the TiO_2_ was located on the surface of the PSt particles, forming a core-shell structure with a thin shell of TiO_2_ covering the PSt core particles. Nevertheless, a careful examination of Figure 1A revealed that tiny TiO_2_ particles, with size of about 10 × 4 nm, as shown in the inserted picture with enlarged magnification at the left bottom corner of Figure 1A, were present as gray and meshy materials between the spherical particles. This suggested that TiO_2_ was not fully deposited on the PSt particles. In contrast, the particles in Figure 1B, prepared using the anionic PSt particles with surface treated with KH550, were of high cleanness. There was no trace of TiO_2_ presence between the spheres, indicating that the *in situ* formed TiO_2_ in this sample was entirely deposited on PSt particles.

It is commonly accepted that polymer particles bearing positive charges on their surface are propitious to SiO_2_ deposition [19]. This was also confirmed in one of our previous works where both anionic and cationic PSt particles were used to prepare PSt composite particles [7]. Comparing Figure 1A to Figure 1B, it is obvious that a much better TiO_2_ deposition was achieved on the PSt particles shown in Figure 1B, i.e., TiO_2_ deposition was better achieved on the particle surface modified with KH550 than on the original cationic particles prepared with DMHB. Although KH550-converted PSt particles were of +10.8 mV in zeta potential, a value lower than +19.9 mV was detected in the cationic PSt particles prepared using DMHB. This indicates that positive charges on PSt particles may not be the main factor to promote the TiO_2_ deposition and, therefore, the formation of PSt/TiO_2_ core-shell structure because the use of the cationic particles converted from anionic ones using KH550 with a lower cationic surface charge seemed more pragmatic than those prepared with cationic DMHB with a higher cationic surface charge. KH550 has been widely applied as a coupling agent, while its amine groups interact with the negative charges on PSt particles, causing KH550 to be densely packed on the surface of PSt particles. Its ethoxy groups are prone to hydrolysis [23], leading to the formation of silicone-hydroxyl (Si-OH) bonds, which are known to undergo a quick condensation between the hydroxyls, leading to Si-O-Si and Si-O-Ti bridging [24,25]. Obviously, this Si-O-Ti bridging builds up an anchoring of TiO_2_ on PSt particles, which will surely favor TiO_2_ deposition onto PSt particles and enhance the stabilization of the resulting core-shell particles.

### Preparation of hollow TiO_2_ particles and their morphology characterization

**Figure 2:**
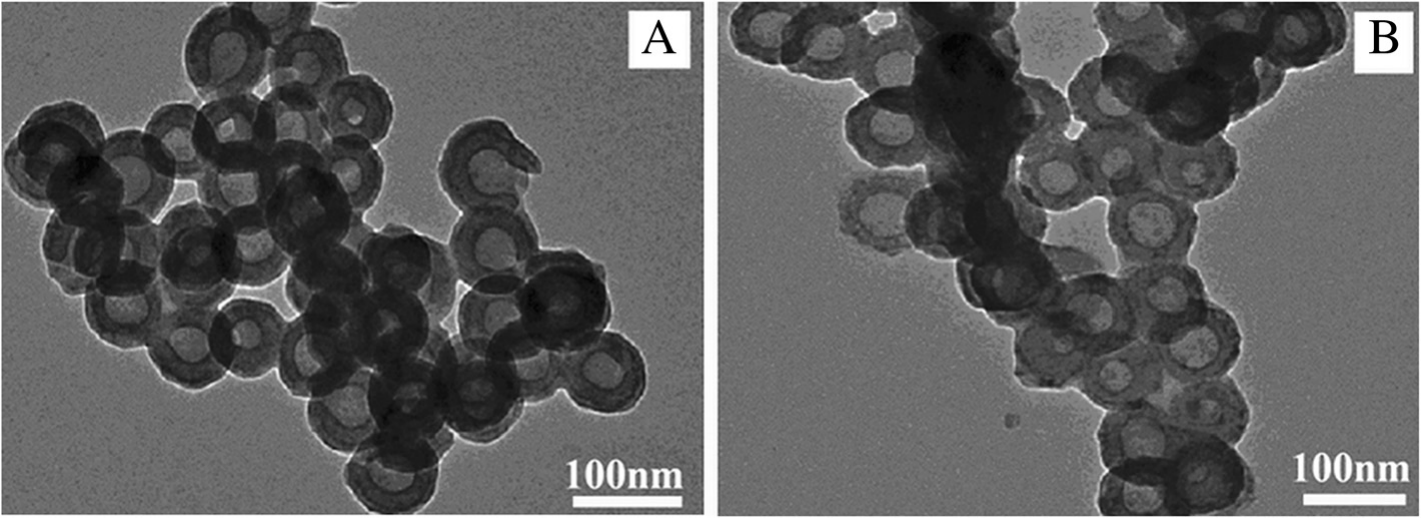
**TEM images of hollow TiO_2_ particles.** Hollow TiO_2_ particles obtained by dissolution of PSt by THF (**A**, sample D) and those by dissolution and preheating of PSt/TiO_2_ (**B**, sample HD).

As revealed above, the PSt/TiO_2_ core-shell structure was well formed with most of the *in situ* formed TiO_2_ located on the PSt particles prepared through KH550 surface modification. All experiments hereafter were done using this type of PSt particles. In Figure 2 are given the TEM pictures of the hollow TiO_2_ particles prepared through a simple THF dissolution (sample D) and those preheated ahead of the THF dissolution (sample HD). It is seen that hollow TiO_2_ particles, with a thickness of 20 to 30 nm for the shell, were well formed in both samples, and no perceptible difference was observed in the two samples. The darker area in Figure 2B was believed to be due to overlapped particles.

**Figure 3:**
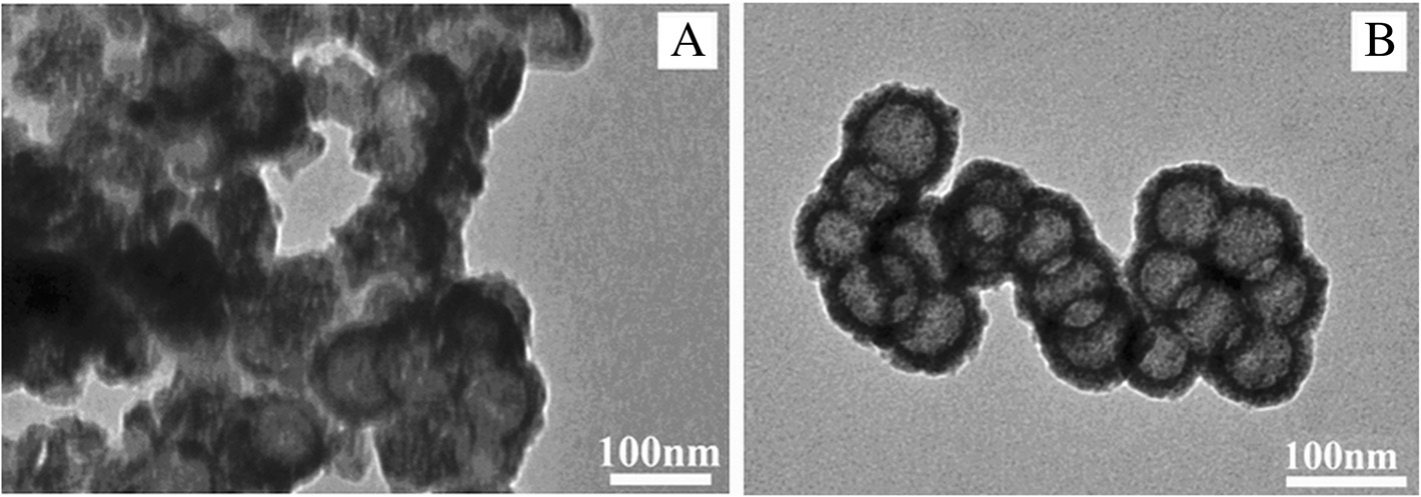
**Hollow TiO_2_ particles.** Hollow TiO_2_ particles obtained by calcination of PSt/TiO_2_ at 600°C (**A**, sample C) and those by calcination and preheating of PSt/TiO_2_ particles (**B**, sample HC).

In Figure 3 are given the TEM pictures of the hollow TiO_2_ particles prepared through the calcination process with (sample HC, Figure 3B) and without preheating (sample C, Figure 3A), as described above. It is clearly seen that, in sample C (the one done through simple calcinations), a great part of the TiO_2_ particles were broken, most of the TiO_2_ particles were aggregated, and not many clear hollow particles were observed. It is believed that TiO_2_ aggregation occurred to form large blocks of crystals along with nucleation and crystal growth when TiO_2_ was undergoing calcination with rapid water loss. Hollow TiO_2_ particles at an early stage of the calcination, if present, may also quickly collapse before crystallization is accomplished. In contrast, a much better TiO_2_ hollow structure with higher uniformity was observed when calcination at 600°C was preceded by a preheating (Figure 3B, sample HC). This showed the necessity and the importance of preheating in this process.

**Figure 4:**
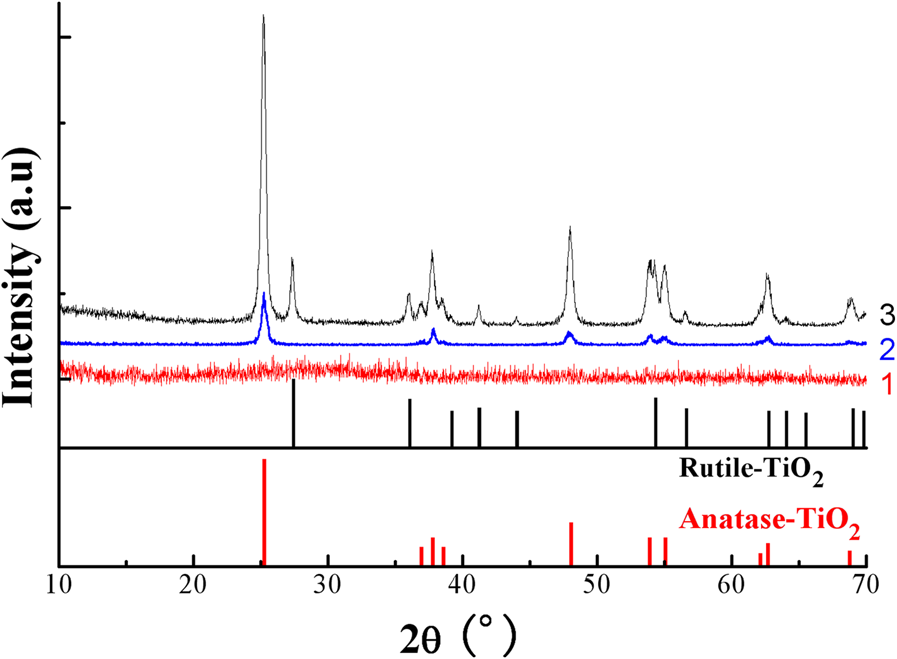
**XRD diffractograms of rutile and anatase TiO_2_ and hollow TiO_2_ particles through different processes.** Curve 1, sample D (simple THF dissolution); curve 2, sample HD (preheating plus THF dissolution); curve 3, sample HC (preheating plus calcination).

To better understand the impact of preheating on the structure of the resulting hollow TiO_2_ particles, selected samples were subjected to XRD structure analyses; the corresponding diffractograms are displayed in Figure 4, along with those of TiO_2_ of rutile and anatase crystals. A remarkable prominence is that there was no crystal of any form in sample D (curve 1 in Figure 4), prepared through simple dissolution of PSt by THF, and TiO_2_ was amorphous by 100%. For sample HD (curve 2 in Figure 4), processed also by PSt dissolution by THF but with preheating, the diffractogram (curve 2 in Figure 4) demonstrates that the formation of anatase phase indeed took place, the characteristic peaks of anatase phase (major peaks: 25.4°, 38.0°, 48.0°, and 54.7°) were all present, and no crystalline phase ascribed to rutile was found, which strongly suggests that the preheating was the only incentive to the formation of anatase crystallization. The size of the crystals was 17 nm based on estimation using Scherrer equation [26].

Comparing the XRD diffractogram of sample HD (curve 2) with that of sample HC (curve 3 in Figure 4), prepared by calcination preceded by a preheating, the following points are to be noted: The crystallized portion in sample HC was significantly higher than that in sample HD. By comparing the diffractograms of sample HD (curve 2) to sample HC (curve 3), on one hand, and to those of rutile and anatase structures, on the other hand, both rutile and anatase structures were formed in sample HC instead of only anatase structure in sample HD. All major peaks of the rutile phase (2*θ* at 27.4°, 36.1°, 41.3°, and 54.4°) appeared in the diffractogram of sample HC. The size of the crystals was 19 nm based on estimation using Scherrer equation [26], about the same as that in sample HD. Based on the relative intensity of the peaks, the proportion of rutile phase was calculated to be 17.0% using a reported equation [27].

### Thermal gravimetry analysis of PSt/TiO_2_ composite and hollow TiO_2_ particles

**Figure 5:**
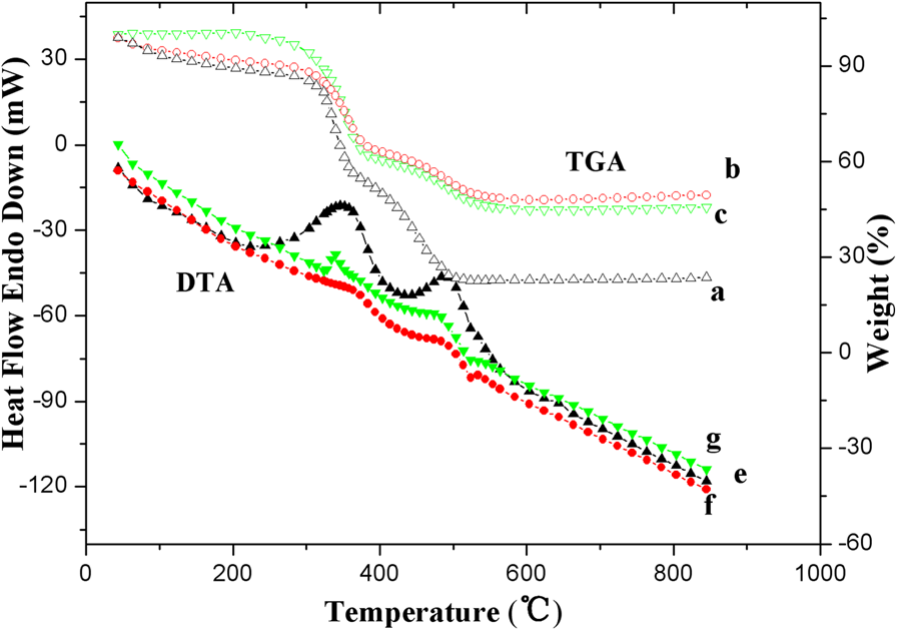
**TGA and DTA curves of PSt/TiO_2_ and TiO_2_ particles.** TGA (curves a, b, and c) and DTA (curves e, f, and g) patterns of as-prepared PSt/TiO_2_ (a and e), hollow TiO_2_ prepared by simple THF dissolution of PSt (sample D: b and f), and those prepared by THF dissolution and preheating (sample HD: c and g).

As-prepared PSt/TiO_2_ composites, hollow TiO_2_ particles obtained via simple dissolution of PSt by THF (sample D) and those via the dissolution with preheating (sample HD), were further subjected to TGA and DTA. Results are displayed in Figure 5.

From the TGA curves, it is clearly seen that an obvious mass loss was detected around 100°C in both PSt/TiO_2_ composite particles (curve a in Figure 5) and sample D (curve b in Figure 5), and this mass loss ended up at 110°C. However, no such mass loss was seen for sample HD; its TGA curve (c) remained unchanged up to about 300°C. It is known from the sample preparation that all processing for both samples, i.e., PSt/TiO_2_ composite particles and sample D, was conducted at 30°C or lower prior to this TGA test, which means that there is moisture remaining in the samples. The mass loss that appeared around 100°C was therefore attributed to moisture evaporation. Keeping the sample processing in mind, it is easy to understand that TiO_2_ in PSt/TiO_2_ composite particles (curve a) and sample D (curve **b**) were completely amorphous, as confirmed in Figure 4 (XRD diffractogram, curve 1). The slight mass decrease from 100°C to 300°C was therefore believed to be owing to gradual crystallization or slow phase transformation in the material, eventually with water loss due to the change from H_4_TiO_4_ to TiO_2_. From 300°C to about 550°C, two significant mass losses were observed. These have been also observed in different reports [28-30]. In one report, both mass losses have been attributed to PSt degradation [28]. In some other studies, the first mass loss, between 300°C and 420°C, was ascribed to thermal degradation of the PSt templates; and the second, from 425°C to 500°C, was ascribed to the decomposition of unhydrolyzed alkoxy groups bonded to titania [29,30]. It should be noted that the magnitude of the first mass loss was about the same for all the three samples, indicating that they contained about the same level of the materials burnt off at this temperature range (300°C to 420°C). As to the second mass loss (425°C to 500°C), it was significantly more pronounced in the PSt/TiO_2_ composite (curve a) than in the other two samples. Knowing that about 40 wt.% of PSt was there in the PSt/TiO_2_ composite (curve a) and that the PSt core template was assumingly removed either by a simple THF dissolution (curve b) or by THF dissolution with preheating (curve c), the second mass loss was attributed to PSt degradation as suggested by Strohm et al. [28], and the first mass loss was attributed to the decomposition of titania-bonded groups, including KH550, hydroxyl, and unhydrolyzed alkoxy groups.

As a homopolymer, PSt itself is known to thermally decompose above 300°C, and full decomposition is usually achieved around 400°C [31]. This may have led to the assignment of the mass loss at 300°C to 420°C to the PSt template in several reports [28-30]. However, thermal decomposition of PSt is dependent on its location [32] and even the size of PSt particles [31]. Jang et al. [32] found that pure PSt nanoparticles started to thermally degrade at about 350°C and completed it at 450°C. However, this degradation shifted to higher temperatures when the PSt particles were incorporated with clay to form nanocomposites. Namely, the thermal stability of PSt was increased because of the presence of inorganic materials in PSt. With 5 wt.% of clay, for instance, the composites degraded between 400°C and 480°C. Knowing the higher content of TiO_2_ in this PSt/TiO_2_ composite, a shift to higher decomposition temperatures (425°C to 500°C) for PSt cannot be excluded. The TGA results were well collaborated with the DTA results shown also in Figure 5, particularly for the PSt/TiO_2_ composite sample, where two heat release peaks appeared, one owing to the decomposition of titania-bonded KH550 and unhydrolyzed alkoxy groups (around 420°C) and the other to the thermal degradation of the PSt templates (around 500°C).

### Catalytic activity of hollow TiO_2_ particles in RB photodegradation

**Figure 6:**
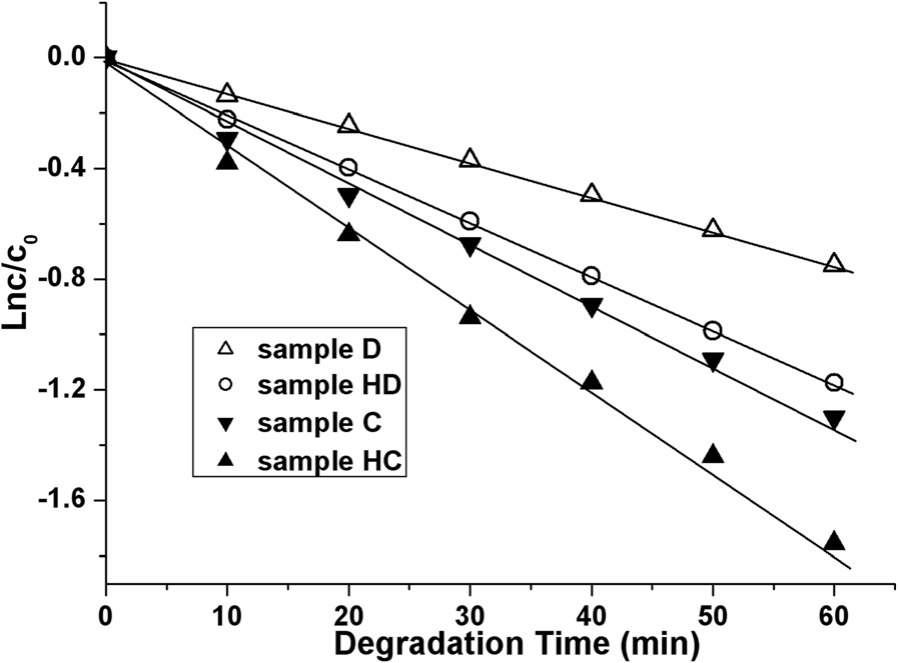
**Catalytic activity of TiO_2_ in RB photodegradation.** White triangle, sample D by simple THF dissolution of PSt; white circle, sample HD by THF dissolution and preheating; inverted black triangle, sample C by simple calcination; black triangle, sample HC by calcination and preheating.

The hollow TiO_2_ particles, as prepared through simple dissolution of PSt by THF (sample D), dissolution with preheating (sample HD), PSt calcination at 600°C (sample C), and calcination with preheating (sample HC), were used in RB photodegradation. Their catalytic activities were tested, and the results are shown in Figure 6.

From Figure 6, it is seen that RB degradation was a first-order reaction. Catalytic activity of the TiO_2_ particles was obviously dependent on their process of preparation. Despite the well-formed hollow structure of the TiO_2_ upon PSt removal, as seen in Figure 2, the samples (D and HD) obtained through THF dissolution demonstrated a lower catalytic activity than those prepared through calcination, regardless of the preheating at 200°C and the partial sintering of sample C as seen in Figure 3. This lower catalytic activity in the samples prepared by dissolution must be owing to the absence of the crystallite structure, either anatase or rutile, as revealed by the data in Figure 4. Comparing sample C (by simple calcination) with sample HC (preheating plus calcination), a much higher catalytic activity was obtained in sample HC, which could be readily attributed on one hand to its well-formed hollow structure (as seen in Figure 3) and to its full crystallization on the other hand, particularly the presence of a certain amount of rutile structure incorporated in anatase structure (as seen in Figure 4) because it is commonly known that the presence of a small amount of rutile phase in anatase TiO_2_ is useful to enhance the particles' catalytic activity [33].

## Conclusions

To prepare PSt/TiO_2_ core-shell nanocomposite particles, the use of cationic PSt particles, particularly those converted from anionic PSt particles via surface modification using KH550, is highly beneficial. For the removal of the PSt core from the PSt/TiO_2_ particles, THF dissolution of the core at 30°C or calcination of the sample at 600°C was used. It was found that hollow TiO_2_ structure was significantly improved when a preheating of the sample at 200°C was applied regardless of the PSt removal process used. Sample characterization using TGA and DTA revealed that PSt degradation observed at about 500°C was preceded by an obvious mass loss and heat release around 400°C owing to the decomposition of titania-bonded unhydrolyzed alkoxy groups. TiO_2_ in the as-prepared PSt/TiO_2_ composites was amorphous, and its phase structure was not changed upon PSt removal through simple THF dissolution. Only anatase structure was formed in the resulting hollow TiO_2_ particles when PSt was removed by THF dissolution preceded by preheating at 200°C, whereas both anatase and rutile crystalline structures were detected in the final hollow TiO_2_ particles when PSt was burned off through calcination preceded by the same preheating. The hollow TiO_2_ particles thus prepared through different processes were used as catalyst in the photodegradation of RB. Results revealed that the highest catalytic activity was obtained with TiO_2_ prepared through calcination combined with preheating ahead of the PSt removal.

## Competing interests

The authors declare that they have no competing interests.

## Authors’ contributions

JW and XZK designed the research. JW and JY performed the experiment. JW analyzed the data and also contributed to manuscript preparation. XZ contributed to the manuscript preparation. XZK finalized the manuscript. All authors read and approved the final manuscript.

## Authors’ information

JW received his BSc degree in analytical chemistry from Shandong Institute of Building Materials Industry (now University of Jinan since 2000) in 1992 and MSc degree in material science from Nanjing University of Technology in 2001. In 2007, he obtained his PhD degree in engineering from China Institute of Atomic Energy. He is currently an associate professor at the University of Jinan. His main research interests focus mainly on preparations of nanoparticle catalysts and the degradation of organic pollutants. He has published over 40 papers up to date.
